# Tumor microenvironment: an evil nexus promoting aggressive head and neck squamous cell carcinoma and avenue for targeted therapy

**DOI:** 10.1038/s41392-020-00419-w

**Published:** 2021-01-12

**Authors:** Ajaz A. Bhat, Parvaiz Yousuf, Nissar A. Wani, Arshi Rizwan, Shyam S. Chauhan, Mushtaq A. Siddiqi, Davide Bedognetti, Wael El-Rifai, Michael P. Frenneaux, Surinder K. Batra, Mohammad Haris, Muzafar A. Macha

**Affiliations:** 1 Functional and Molecular Imaging Laboratory, Cancer Research Department, Sidra Medicine, Doha, Qatar; 2 Department of Zoology, School of Life Sciences, Central University of Kashmir, Ganderbal, Jammu & Kashmir India; 3 Department of Nephrology, All India Institute of Medical Sciences, New Delhi, India; 4 Department of Biochemistry, All India Institute of Medical Sciences, New Delhi, India; 5 Watson-Crick Centre for Molecular Medicine, Islamic University of Science and Technology, Awantipora, Jammu & Kashmir, India; 6 Laboratory of Cancer Immunogenomics, Cancer Research Department, Sidra Medicine, Doha, Qatar; 7 Department of Surgery, University of Miami, Miami, FL USA; 8 Academic Health System, Hamad Medical Corporation, Doha, Qatar; 9 Department of Biochemistry and Molecular Biology, University of Nebraska Medical Center, Omaha, NE USA; 10 Eppley Institute for Research in Cancer and Allied Diseases, University of Nebraska Medical Center, Omaha, NE USA; 11 Buffett Cancer Center, University of Nebraska Medical Center, Omaha, NE USA; 12 Laboratory Animal Research Center, Qatar University, Doha, Qatar

**Keywords:** Head and neck cancer, Cancer microenvironment

## Abstract

Head and neck squamous cell carcinoma (HNSCC) is a very aggressive disease with a poor prognosis for advanced-stage tumors. Recent clinical, genomic, and cellular studies have revealed the highly heterogeneous and immunosuppressive nature of HNSCC. Despite significant advances in multimodal therapeutic interventions, failure to cure and recurrence are common and account for most deaths. It is becoming increasingly apparent that tumor microenvironment (TME) plays a critical role in HNSCC tumorigenesis, promotes the evolution of aggressive tumors and resistance to therapy, and thereby adversely affects the prognosis. A complete understanding of the TME factors, together with the highly complex tumor–stromal interactions, can lead to new therapeutic interventions in HNSCC. Interestingly, different molecular and immune landscapes between HPV^+ve^ and HPV^−ve^ (human papillomavirus) HNSCC tumors offer new opportunities for developing individualized, targeted chemoimmunotherapy (CIT) regimen. This review highlights the current understanding of the complexity between HPV^+ve^ and HPV^−ve^ HNSCC TME and various tumor–stromal cross-talk modulating processes, including epithelial–mesenchymal transition (EMT), anoikis resistance, angiogenesis, immune surveillance, metastatic niche, therapeutic resistance, and development of an aggressive tumor phenotype. Furthermore, we summarize the recent developments and the rationale behind CIT strategies and their clinical applications in HPV^+ve^ and HPV^−ve^ HNSCC.

## Introduction

Head and neck squamous cell carcinoma (HNSCC) is the 6th most common cancer worldwide, with an annual incidence of more than 800,000 new cases and 350,000 deaths.^1–3^ Clinically, pathologically, phenotypically, and biologically, HNSCC is a heterogeneous disease of the oral cavity, oropharynx, hypopharynx, larynx, and paranasal sinuses.^4,5^ Oral squamous cell carcinoma (OSCC) is the major subtypes of HNSCC and accounts for two-thirds of the cases in developing countries. While tobacco and alcohol consumption are responsible for ~75% of HNSCC cases, recently, a substantial increase in human papillomavirus (HPV)-associated oropharynx cancers (OPC)^6^ in the Western world has been observed which is expected to surpass cervical cancers by 2020 in the USA.^7^ In contrast to the etiological role of tobacco smoking in Western countries,^8^ the use of smokeless tobacco (ST) products like pan masala, gutkha, and betel quid are the major risk factors in Asian countries, including India.^9–12^ Other etiological factors such as exposure to radiation,^13^ wood dust,^14^ asbestos,^15^ salted foods,^14^ poor oral hygiene,^16^ and Epstein–Barr virus (EBV) infection^17^ also increase the risk of HNSCC.

HNSCCs are mostly diagnosed at an advanced stage with locally advanced (LA) or distant metastasis (DM). Despite multimodality therapeutic interventions which include surgery, radiotherapy (RT), chemotherapy (CT), and/or immunotherapy (IT), a majority (40–60%) of the LA tumors ultimately display recurrence/local progression. Treatment of metastatic and recurrent (R/M) HNSCC tumors with palliative CT also displays poor prognosis. The complete understanding of the HNSCC tumor biology might help us overcome the low therapeutic response of HNSCCs and aid in developing therapeutic strategies with minimal inherent or acquired resistance. Therefore, understanding the HNSCC biology and identifying novel therapeutic targets for effective management of this malignancy is the dire need.^18^

Most of the previous studies were focused on targeting only cancer cells. However, recent studies have shown that noncancerous cells surrounding the tumor and extracellular matrix (ECM) proteins, which together form the TME, play a critical role in tumorigenesis, the evolution of aggressive tumors, and the promotion of resistance to therapy.^19,20^ The TME is enriched with various growth factors, intermediate metabolites, nutrients, hormones, growth factors, including chemokines, cytokines, and immune modulators that are secreted by both the tumor and stromal cells. These factors promote the clonal selection of aggressive cells and the acquisition of many of the hallmarks of cancer.^21,22^ Thus TME provides a permissive environment for tumor progression, metastasis, and development of resistance.^23^

Interestingly, recently developed therapeutic strategies targeting both the cancer cells and components of the TME have shown increased efficacy and improved patient prognosis.^24^ In this review, we discuss the current understanding and complexity of TME in both HPV^+ve^ and HPV^−ve^ HNSCC, and tumor–stromal cross-talk modulating processes, including EMT, anoikis resistance, angiogenesis, immune surveillance, metastatic niche, therapeutic resistance, which together contribute to the development of aggressive tumors and resistance to therapy. We also summarize the recent developments and the rationale behind CIT strategies and their clinical applications in HNSCC.

## HNSCC tumor microenvironment

**Fig. 1:**
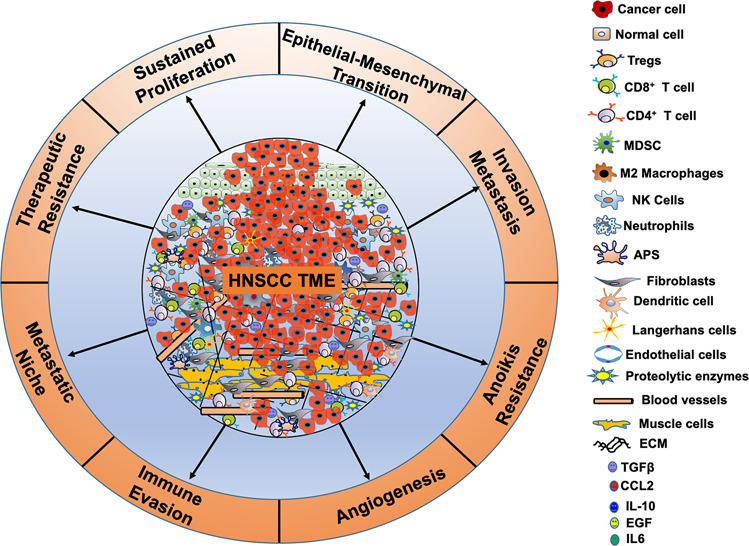
Head and neck squamous cell carcinoma (HNSCC) TME is a complex ecosystem. HNSCC TME is a complex ecosystem consisting of a fabricated network of tumor cells surrounded by non-tumor cells, including cancer-associated fibroblasts (CAFs), endothelial cells (EC), adipocytes, neuroendocrine cells, blood and lymphatic vascular cells, muscle cells, infiltrating immune cells ((T cells, B cells, natural killer cells (NK cells), neutrophils, dendritic cells (DC), Langerhans cells (LCs), macrophages, and myeloid-derived suppressor cells (MDSCs)]. In addition, stromal components, including extracellular matrix (ECM) proteins (collagen, fibronectin, elastin, laminin, and tenascin), intermediate metabolites, nutrients, hormones, growth factors, etc. are the crucial components of HNSCC TME. The complex cross talk between the tumor and the stromal components regulates cell growth, epithelial–mesenchymal transition (EMT), invasion and metastasis, anoikis resistance, angiogenesis, metastatic niche, immune surveillance, and therapeutic resistance making HNSCC tumors very aggressive

Cancer was previously deemed a mass of undifferentiated tumor cells without considering the surrounding stromal cells in the microenvironment. In HNSCC, the TME represents a highly complex ecosystem of cellular and noncellular components. The cellular constituents include genetically altered stromal cells such as cancer-associated fibroblasts (CAFs), endothelial cells (EC), adipocytes, neuroendocrine cells, blood and lymphatic vascular cells,^25^ and infiltrating immune cells ((T cells, B cells, natural killer cells (NK cells), dendritic cells (DC), macrophages, myeloid-derived suppressor cells (MDSCs)) (Fig. 1).^26–28^ The noncellular components of the TME include ECM proteins such as collagen, fibronectin, elastin, laminin, and tenascin and physical and chemical parameters, such as pH, oxygen tension, interstitial pressure, and fluid flux. Recent studies have shown that stromal cells provide intermediate metabolites, nutrients, hormones, cytokines/chemokines, and growth factors to tumor cells to support their proliferation, invasion, metastasis, and survival.^29–32^ They also help recruit CAFs,^33^ tumor-promoting immune cells, inflammatory cells, and help escape immune recognition,^34^ thereby providing a permissive environment for tumor progression, metastasis, and development of resistance to therapy.^23^ These studies suggest that TME dictates aberrant tissue function and plays a critical role in the subsequent evolution of more advanced malignancies.^35^

HNSCC TME is infiltrated with lymphocytes (TILs) and their subsets such as CD8^+^ cytotoxic T cells, CD4^+^ helper T cells, CD163^+^ and CD68^+^ macrophages and MDSCs, CD57^+^ NK cells, FOXP3^+^ T-regulatory (Tregs) cells^36,37^ attributing prognostic value to TILs. Besides, substantial infiltration of pro-inflammatory immune cells, such as tumor-associated macrophages (TAM), natural killer (NK) cells, and CD8^+^ T cells, HNSCC TME is immunosuppressive.^34^ Expression of pro-inflammatory mediators, such as IL-1α, IL-1β, IL-6, IL-8, TNF-α, TGF-β, granulocyte–macrophage colony-stimulating factor (GM-CSF), vascular endothelial growth factor (VEGF), monocyte chemoattractant protein 1 (MCP-1), RANTES (CCL5), and prostaglandin E2 (PGE2) are found to be upregulated in the premalignant lesions of HNSCC^38,39^ and are involved in progression and metastasis. Similarly, overexpression of alpha-smooth muscle actin (α-SMA), a marker for cancer-associated macrophages (CAFs), is observed in premalignant lesions compared to normal epithelia.^40,41^ A study using a 4-nitro-quinoline-2-oxide (4-NQO) induced mouse model of HNSCC found elevated levels of inflammatory Th1, TC1, Th17 cells, and increased expression of IL-17, IL-23 in premalignant lesions. Further, increased infiltration of Tregs along with the downregulation of IL-23 and upregulation of TGF-β in the tumor tissues was also observed Table 1. These results are suggestive of an immune-stimulatory microenvironment in the premalignant stages compared to an immunosuppressive TME of the established tumors.^42^ Similarly, increased IL-6 and TNF-α expression was observed in the saliva of HNSCC patients.^43^

**Table 1:** Differences between HPV^+ve^ and HPV^−ve^ HNSCC TME

Serial No.	Cell type	HPV status	Levels in tumor	Protein expression/factors secreted	Prognosis	Ref.
1	CD3^+^	HPV^+ve^	Higher	IFN-γ	Better	^64^
2	CD4^+^		Higher	IFN-γ, TNF-α	Better	^65^
3	CD8^+^		Higher	IFN-γ, IL-17	Better	^64^
4	CD4^+^/CD8^+^ ratio		Lower		Better	^74^
5	CD4^+^CD25^+^ Tregs		Higher	IL-10, IL-12, IL-35, TGF-β, VEGF	Better	^71^
6	CD56^+^ NK cells		Higher	IFN-γ,	Better	^71^
7	M1 macrophages		Lower	IL-12, IL-23, TNF-α, CCL5, CXCL9, CXCL5, CXCL10	Better	^74^
8	M2 macrophages		Higher	TGF-β, IL-13, IL-4, IL-10, MMP9, CCL2, CCL5, CXCL2, CXCL8, MIF, EGF, VEGF, ROS, arginase-1	Poor	^72^
9	MDSCs		Higher	iNOS, NO, ROS, MMP9, PD-L1, arginase-1	Poor	^70^
10	CD45^+^ lymphocytes		Higher		Better	^54^
11	CD19^+^/CD20^+^ B cells		Higher		Better	^54^
12	Tumor-infiltrating APCs		Higher	--	Better	^70^
13	Langerhans cells		Higher	--	Better	^70^
14	Chemokines		Higher	IL-10, CCL2, EGF	Poor	^55,56^
15	Cytotoxic mediators		Higher	Granzyme A, granzyme B, perforin	Better	^53,76^
16	Protein molecules		Lower	E-cadherin, MIP-3, IFN-γ, MHC1 CD1d	Poor	^79,81,85^
			Higher	LAG3, PD-1, TIGIT, TIM3, CD39, COX2, PD-L1, PD-L2	Poor	^202,238^
17	Metabolism Core Periphery	HPV^-ve^	Higher Higher	Aerobic glycolysis OXPOS	Poor	^28^
18	Metabolic mediators Core Periphery		Lower Higher	GLUT1, MCT1, COX5B, and LDHB	Poor	^28^

*Tregs* T regulatory cells, *NK cells* natural killer cells, *MDSCs* myeloid-derived suppressor cells, *APC* antigen-presenting cells, *MIP-3* macrophage inflammatory protein 3, *Tim-3* T-cell immunoglobulin and mucin domain-3, *LAG3* lymphocyte-activation gene 3, *PD-1* programmed death-1, *TIGIT* T-cell immune receptor with Ig and ITIM domains, *GLUT1* glucose transporter-1, *LDHB* lactate dehydrogenase-B, *MCT1* monocarboxylase transporter 1, *COX2* cyclooxygenase-2, *COX5B* cyclooxygenase 5B, *OXPHOS* oxidative phosphorylation

## Tumor microenvironment differs in HPV^+ve^ and HPV^−ve^ HNSCC

**Fig. 2:**
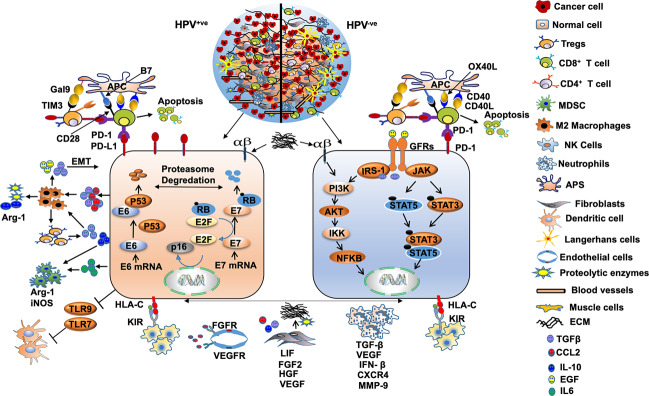
TME of HPV^+ve^ and HPV^−ve^ HNSCC tumors. The HPV^+ve^ (left) and HPV^−ve^ (right) HNSCC TME have different cell composition and differential tumor–stromal cross talk. The HPV^+ve^ tumors show increased infiltration of CD3^+^, CD4^+^, CD8^+^ cells, CD56^dim^ NK cells, APCs, MDSCs, DCs, and lower Tregs infiltration; however, the converse is true for HPV^−ve^ tumors. HPV^−ve^ tumors have increased the infiltration of M1 macrophages and Langerhans cells. As compared to HPV^−ve^ tumors, HPV^+ve^ tumors have increased secretion of various cytokines, chemokines, and growth factors, including IL-10, CCL2, IL-6, TGF-β, TNF-α, and EGF that provide a growth advantage to the tumor cells and induce immunosuppression. Increased secretion of TGF-β and CCL2 by the HPV^+ve^ cells promotes macrophage differentiation to pro-tumorigenic M2 that stimulates Tregs. In turn, Tregs induce LT CD8^+^ exhaustion and apoptosis through PD-L1-PD-1 interaction. M2 secretes IL-6 and IL-10 that stimulate MDSCs, TGF-β and EGF induces epithelial-to-mesenchymal transition (EMT) on cancer cells. While the HPV^+ve^ tumors have enhanced OXPHOS at the core and aerobic glycolysis in the tumor periphery (blue zone), the opposite is true for HPV^−ve^ tumors and has more deposition of lactate known to suppress the immune cells

The HPV^−ve^ tumors mostly occur in the tongue, buccal mucosa, hard palate, lips, while the HPV^+ve^ tumors are commonly observed in the palatine and lingual tonsillar region.^44,45^ In addition, HPV^−ve^ OPC and non-OPC patients are typically older when compared to HPV^+ve^ OPC.^46–48^ While TP53, CCND1, CDKN2A, FGFR1, MLL2, CUL3, NSD1, PIK3CA, and NOTCH are highly mutated in HPV^−ve^ HNSCC,^49^ the higher mutational incidence of DDX3X, FGFR2, FGFR3 PIK3CA, KRAS, MLL3, and NOTCH-1 is observed in HPV^+ve^ HNSCC.^50^ Interestingly, increased cancer stem cells (CSC) population with higher expression of CSC markers, OCT4, SOX2, KLF4, and BIM1 were reported in HPV^−ve^ OPCs^51^ and associated with a lower response to CRT and worse patient survival.^52^ Further comprehensive analysis of The Cancer Genome Atlas (TCGA) data has established the immunologically active nature of HNSCC tumors.^53^ However, further characterization of these tumors revealed HPV^−ve^ as immunologically cold tumors as compared to their HPV^+ve^ counterparts. Specifically, HPV^+ve^ and HPV^−ve^ OPC tumors have more TILs, Tregs (CD3^+^ and CD8^+^), exhausted CD4^+^ and CD8^+^ PD-1^+^ T cells, NK cells, and B cells^54^ (Fig. 2 and Table 2). Increased infiltration of these TILs is associated with increased production of CCL17, CCL21, IL-10, IL-17, IL-21, TNF-α, and IFN-γ, thereby suggesting an HPV-specific T-cell response that supports favorable OS in HPV^+ve^ HNSCC.^55–60^ Other studies revealed significantly more numbers of FOXP3^+^ Tregs in the stromal and intraepithelial compartments of HPV^+ve^ HNSCC tumors compared to HPV^−ve^ tumors.^61–63^ While some of these studies reported an association of Tregs infiltration with better overall survival (OS) and disease-free survival (DFS),^54,62^ others observed inferior recurrence-free survival (RFS) and OS with Tregs infiltration.^61,63^ Higher CD3^+^ and CD8^+^ T-cell infiltration was also reported in HPV^+ve^ OPCs compared to HPV^−ve^ tumors,^64^ and increased CD8^+^ T-cell infiltration was strongly associated with improved OS and locoregional control (LRC).^54,62^ Similarly, higher CD4^+^ TILs in HPV^+ve^ OPC were associated with better prognosis.^65^ The presence of HPV16 and E7-specific T-cell and circulating T lymphocytes in HPV^+ve^ OPCs,^66,67^ and their correlation with survival outcome is also documented.^68^ Oncogenic E6 and E7 HPV proteins function as tumor-associated antigens and activate CD8^+^ cytotoxic T lymphocytes (CTLs) via DCs.^69^ However, HPV E7 has been shown to decrease the expression of Toll-like receptor-9 (TLR9), which are involved in the activation of DCs. In contrast, higher tumor intraepithelial infiltration of MDSCs was observed in HPV^+ve^ HNSCC tumors compared to HPV^−ve^ tumors.^70^ Tumor-associated macrophages (TAMs) are essential for tumorigenesis and controlling angiogenesis, invasion and migration, EMT, intravasation and extravasation, and immunosuppression.^71^ Furthermore, increased CD68^+^ macrophage infiltration in HNSCC has been reported to be associated with lymph node metastasis,^72^ shorter RFS and OS.^73^ While increased M2 macrophage infiltration in HNSCC TME has been shown to contribute to local and systemic immunosuppression,^72^ increased M1 macrophage levels in HPV^+ve^ HNSCC patients showed favorable prognosis,^74^ possibly due to increased M1/M2 ratio. In contrast, increased macrophage recruitment in HPV^+ve^ HNSCC tumors compared to HPV^−ve^ tumors was reported to be associated with shorter RFS and OS.^75^ In addition to TAMs, significantly increased CD56^+^ NK cells and increased granzyme B expression were reported in HPV^+ve^ OPCs compared to HPV^−ve^ counterparts^53,76^ that correlate with improved OS.^53,76^ Langerhans cells (LCs) are the antigen-presenting cells (APCs) of the immune system and decreased LCs density represents compromised immune surveillance. While the progressive increase of LC infiltration was observed in the transition of normal phenotype to dysplasia and eventually to cancers,^77^ higher intraepithelial infiltration of LCs was observed in HPV^−ve^ HNSCC tumors than HPV^+ve^ tumors.^77^ Importantly, increased LC infiltration was associated with improved RFS and OS.^77^ Though the underlying mechanisms are still unclear, E6- and E7-associated decrease in E-cadherin and macrophage inflammatory protein 3 (MIP-3) has been shown to impair LC recruitment and retention.^78,79^

**Table 2:** Clinical trials targeting HPV^+ve^ and HPV^-ve^ HNSCC TME

Serial No.	Antibodies/drugs used	Target	HNSCC patients	Status	Phase	Trial ID number
**1**	Durvalumab (MEDI4736) monotherapy	PD-L1	HPV^+ve/−ve^ PR R/M	Active, not recruiting	II	NCT02207530
**2**	Durvalumab (MEDI4736) + tremelimumab (anti-CTLA-4 Ab) vs Durvalumab	PD-L1 and CTLA-4	R/M	Active, not recruiting	II	NCT02319044
3	Durvalumab (MEDI4736) with/without tremelimumab *vs* SOC (docetaxel + methotrexate + 5FU + cetuximab (EAGLE)	PD-L1, CTLA-4, and EGFR	PD-L1^+ve^/^−ve^ R/M	Active, not recruiting	III	NCT02369874
4	Durvalumab with/without tremelimumab vs cetuximab + cisplatin + carboplatin + 5FU	PD-L1, CTLA-4, and EGFR	R/M	Active, not recruiting	III	NCT02551159
5	Durvalumab + RT + cisplatin vs RT + Cisplatin + placebo (ADHERE)	PD-L1	HPV^-ve^	Not yet recruiting	III	NCT03673735
6	Avelumab + IMRT + cetuximab (anti-EGFR Ab) vs SOC (IMRT + cisplatin) (REACH)	PD-L1 and EGFR	LA	Recruiting	III	NCT02999087
7	Avelumab + cetuximab + palbociclib	PD-L1, EGFR, and CDK4/6	R/M HNSCC	Recruiting	I	NCT03498378
8	Atezeolizumab + bevacizumab (anti-VEGF Ab)	PD-L1 and VEGF	HPV^+ve^ or EBV^+ve^ HNSCC and other solid tumors	Recruiting	II	NCT03074513
10	Pembrolizumab (MK-3475)	PD-1	High risk for recurrence	Recruiting	II	NCT02841748
11	Pembrolizumab (MK-3475-012) (KEYNOTE-012)	PD-1	HPV^+ve/-ve^ HNSCC and others	Active, not recruiting	I	NCT01848834
12	Pembrolizumab + RT vs RT + cisplatin	PD-1	LA	Recruiting	II	NCT03383094
13	Pembrolizumab (MK-3475) + platinum-based chemotherapy and 5FU with or without cetuximab (KEYNOTE-048)	PD-1	R/M	Active, not recruiting	III	NCT02358031
14	Pembrolizumab + cetuximab	PD-1 and EGFR	R/M	Recruiting	II	NCT03082534
	Pembrolizumab (MK-3475) vs SOC (methotrexate + docetaxel + cetuximab) (KEYNOTE-040)	PD-1 and EGFR	R/M	Ongoing	III	NCT02252042
15	Pembrolizumab (anti-PD-1 Ab, MK-3475)	PD-1	RD Following definitive CRT	Recruiting	II	NCT02892201
17	Pembrolizumab + cisplatin and IMRT	PD-1	LA	Recruiting	II	NCT02777385
18	Pembrolizumab (MK-3475) + RT + cisplatin (MK-3475-412/KEYNOTE-412)	PD-1	LA	Active, not recruiting	III	NCT03040999
19	Nivolumab vs cetuximab/methotrexate/docetaxel (CheckMate 141)	PD-1	R/M HNSCC	Active, not recruiting	III	NCT02105636
20	Nivolumab (anti-PD-1 Ab) + RT (REPORT)	PD-1	R	Recruiting	I/II	NCT03317327
21	Nivolumab + STBR vs nivolumab	PD-1	Metastatic	Active, not recruiting	II	NCT02684253
22	Nivolumab + IMRT/cisplatin vs nivolumab	PD-1	LA	Active, not recruiting	II	NCT02764593
23	Nivolumab (MDX1106,BMS-936558) vs therapy of investigator’s choice (methotrexate + cetuximab + docetaxel) (CheckMate 141)	PD-1	PR R/M	Active, not recruiting	III	NCT02105636
24	Nivolumab (BMS-936558) + RT vs cetuximab + RT vs nivolumab + cisplatin + RT vs cisplatin + RT	PD-1 and EGFR	LA	Active, not recruiting	III	NCT03349710
25	Ipilimumab + cetuximab + IMRT	CTLA-4 and EGFR	Stage III-IVB	Active, not recruiting	Ib	NCT0186043, NCT01935921
	Ipilimumab + IMRT vs ipilimumab+ nivolumab	CTLA-4 and PD-1	Stage III-IVB	Active, not recruiting	Ib	NCT01860430
26	Nivolumab + varlilumab (anti-CD27)	PD-1 and CD27	LA/R	Completed	III	NCT02335918
28	Nivolumab (BMS-936558) + ipilimumab (BMS-734016) vs SOC (cetuximab + carboplatin + platinum + 5FU)	PD-1, CTLA-4, and EGFR	R/M	Active, not recruiting	III	NCT02741570
	Nivolumab + ulocuplumab (BMS-936564)	PD-1 and CXCR4	LA/M	Terminated	I/II	NCT02472977
29	Anti-OX40 antibody (MEDI6469)	OX40	LA	Active, not recruiting	Ib	NCT02274155
30	Durvalumab (MEDI4736) + agonistic anti-OX40-antibody (MEDI6383) vs MEDI6383	PD-1 and OX40	R/M	Completed	I	NCT02221960
31	Nivolumab (antiPD1 Ab, BMS-936558) + lirilumab (anti-KIR Ab, BMS-986015) vs nivolumab + ipilimumab (anti-CTLA4 Ab)	PD-1, KIR, and CTLA-4	LA/R HNSCC and solid tumors	Active, not recruiting	I/II	NCT01714739
32	Nivolumab vs nivolumab + ipilimumab vs nivolumab + relatlimab (anti-LAG3 Ab, BMS-986016) (Checkmate 358).	PD-1, CTLA-4, and LAG3	EBV^+ve^	Recruiting	I/II	NCT02488759
33	Sunitinib (SU11248)	VEGFR, PDGFR, c-kit, RET, CSF-1R, and FLT3	R/M	Completed	II	NCT00387335
34	Sorafinib + cetuximab vs cetuximab	VEGFR, PDGFR, Raf, and c-kit	R/M	Completed	II	NCT00939627
35	Dasatinib (BMS-354825)	SRC	R/M	Completed	II	NCT00507767

*DCR* disease control rate, *DES* dose escalation study, *LA* locally advanced, *R* recurrent, *M* metastatic, *OS* overall survival, *PFS* progression-free survival, *PR* platinum refractory, *ORR* objective responsive rate, *SOC* standard of care, *IMRT* intensity-modulated radiation therapy, *DLT* dose-limiting toxicity, *EFS* event-free survival, *MTD* maximum-tolerated dose, *RD* residual disease

The role of tumor suppressor p53 in promoting immune infiltration in HPV^+ve^ HNSCC is well known; however, the underlying mechanisms of this disparate immune infiltration is yet to be established. High incidence of p53 mutations in HPV^−ve^ HNSCCs may partially explain these observations.^80^ While this and other studies suggest immune-rich TME in HPV^+ve^ tumors, E6 and E7 proteins evade immune response through downregulation of IFN-γ^81^ and inhibition of the NF-κB pathway,^82,83^ resulting in persistent HPV infection. Furthermore, increased IL-10 and TGF-β secretion by the Tregs suppressed immune cells prevent HPV clearance.^79,84^ Besides, HPV E5 protein leads to the downregulation of MHC1 CD1d and helps protect the recognition of infected cells from NK cell.^85^ In addition to the immunological differences between HPV^+ve^ and HPV^−ve^ tumors, recent studies have shown differential metabolic compartmentalization inside these tumors.^28^ While HPV^+ve^-associated tumors display increased oxidative phosphorylation (OXPHOS) in the tumor core and higher rate of aerobic glycolysis, the inverse is true for HPV^−ve^ tumors.^55^ This differential metabolism is associated with increased centrally localized staining of glucose transporter-1 (GLUT1), lactate dehydrogenase-B (LDHB), monocarboxylase transporter 1 (MCT1), and cyclooxygenase 5B (COX5B) in HPV^+ve^ tumors, but more peripheral staining along with the higher concentration of lactic acid in HPV^−ve^ tumors.^55^ Furthermore, differential gene expression and lactate location is correlated with intratumoral abundance of cytotoxic CD8^+^ T-cell infiltration^55^ and peritumoral presence of pro-inflammatory cytokines, CD8^+^ IFN-γ/IL-17^+^ TL, and myeloid dendritic cells.^86^ These studies suggest that immune-metabolic cross talk and HPV^−ve^ tumors favor more immunosuppressive TME with decreased cytotoxic tumor-infiltrating T cells and increased Tregs cells.

Though many diagnostic and prognostic biomarkers have been identified, only a few are validated for use in clinical practice for HNSCC subtypes. Concerning HPV^+ve^ OPC, PCR-based HPV DNA/E6 or E7 mRNA detection,^87^ and serological markers including antibodies against HPV16 capsid protein L1, or oncoproteins E6 and E7 are routinely used.^88–91^ In addition, p16 overexpression is an important surrogate biomarker for HPV infection and is associated with favorable outcomes for HPV^+ve^ OPC.^92^ It is interesting to mention that p16-positivity has been included in the recent WHO TNM classification for OPCs. As compared to HPV^−ve^ tumors, HPV^+ve^ OPCs have higher CD3^+^ and CD8^+^ TILs,^64^ suggesting a more antitumor immune response.^54,62^ Based on these studies, immunological biomarkers or immunoscore with the diagnostic and predictive outcome has been developed.^93^ The immunoscore quantifies CD3^+^ and CD8^+^ TILs in the tumor core and the invasive margin of resected tumors.

Furthermore, tumor mutational burden (TMB) is being currently used as a predictive marker of response to ICI’s.^94^ Higher TMB in patients with solid tumors was associated with better outcomes after treatment with a combination of anti-CTLA-4 and anti-PD-L1/PD-1 compared to monotherapy.^95^ Interestingly, in HNSCC, patients with higher TMB showed better response to anti-PD-L1/PD-1 treatment.^96,97^ Though differences in TMB has been reported in HPV^+ve^ and HPV^−ve^ HNSCC with more TMB observed in HPV^−ve^ tumors,^50^ the clinical significance of these genomic alterations requires further validation in clinical settings. Similarly, the presence of EBV DNA in the plasma has a predictive and prognostic role in nasopharyngeal carcinoma (NPC). Future studies should be focused on developing biomarkers based on molecular, immunological, and TMB in combination with imaging techniques for effective diagnosis and prognosis of HNSCC subtypes.

## TME promotes aggressive HNSCC tumors

The role of TME has a more profound influence on the growth and metastasis of HNSCC. Secretion of growth factors, cytokines, chemokines, hormones, and other chemical inducers by both the stromal and cancer cells activate a vast array of signaling pathways like EGFR, mitogen-activated protein kinases (MAPKs), mammalian target of rapamycin (mTOR), signal transducer and activator of transcription (STAT), Janus kinase (JAK), phosphoinositide 3-kinase (PI3K), and protein kinase C (PKC) pathways.^98,99^ These pathways are involved in regulating cell cycle, growth, differentiation, EMT, anoikis resistance, invasion and metastasis, angiogenesis, apoptosis, stemness in cancer cells, immune surveillance, metastatic niche, and the therapeutic response, thus making tumors more aggressive.

### Role of TME in epithelial–mesenchymal transition in HNSCC

Epithelial-to-mesenchymal transition (EMT) is a crucial process whereby tumor cells lose epithelial characteristics and acquire mesenchymal properties changing cellular morphology, altered cell–cell and cell–matrix adhesion with more invasion and metastatic properties.^100,101^ Several proteins, including Snail, Slug, TWIST, and SMAD nuclear interacting protein 1 (SNIP1), play essential role(s) in promoting EMT by upregulating α-SMA, vimentin, fibroblast-specific protein 1 (FSP-1) and N-cadherin and downregulating E-cadherin and β-catenin expression in HNSCC.^102^ Many recent studies have conclusively established the involvement of TME in HNSCC EMT. Studies have shown that HNSCC cells recruit and educate monocytes into TAMs via the CCL2/CCR2 axis, which secrete migration inhibitory factor (MIF), EGF, and TGF-β, which in turn promote EMT in HNSCC.^103^ The TAM-secreted MIF recruit neutrophils that secrete CCL4, IL-8, and TNF-α to induce EMT and invasive behavior of HNSCC cells.^27^ In addition, MDSCs in HNSCC TME secrete MMP9, EGF, bFGF, IL-1β, and TGF-β, which play a critical role in inducing the EMT in HNSCC.^104,105^ IL-1β secreted by MDSCs reduces the expression of E-cadherin by increased binding of Zeb1 to the Ebox element on its promoter as well as through COX2-dependent upregulation of Snail.^106,107^ Secretion of CXCL12 by CAFs through CXCR4 signaling pathway activation^108,109^ induces EMT in HNSCC,^110,111^ and promotes tumor progression and metastasis.^112^ Besides, CAF-secreted molecules like endothelin-1, CCL7, TGF-β1 via the TGF-β/Smad signaling pathway, and SDF1 via activation of the PI3K-Akt/PKB signaling pathway also promote EMT in HNSCC cells.^110,113–115^ Furthermore, HNSCC secreted IL-1 has been shown to promote TGF-β and HGF production by CAFs known inducers of EMT. Additionally, IL-6 secreted by tumor and many stromal cells through upregulation of Snail via the STAT3 pathway, and stabilizing Twist via casein kinase 2 (CK2) promote HNSCC EMT.^116^ Hypoxia is the vital component of TME and a vital contributor to metastasis and has been shown to induce EMT. HIF-1α, an essential hypoxia-regulated gene, has been reported to control the expression of all the EMT regulators, including Snail, Slug, TWIST, and SNIP1.^27^ HIF-1α increased Twist expression by binding to its HRE proximal promoter element. Hypoxia also promotes EMT in HNSCC by NOTCH signaling activation^117^ and by regulating the metadherin (MTDH) loop.^118^ Through cooperative regulation of Twist and Bmi1, hypoxia represses E-cadherin, and p16^INK4a^ expression to promote EMT in HNSCC.^119^ Interestingly, by increasing the expression of Twist, and reducing that of cyclin D1 and p16^INK4^, EMT promotes dormancy of disseminated tumor cells (DTCs) within the niche.

### Role of HNSCC TME in anoikis resistance

**Fig. 3:**
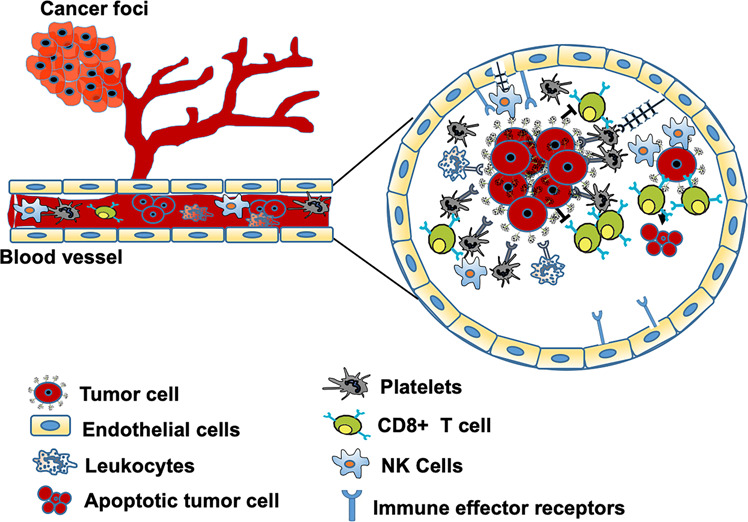
Formation of Emboli and anoikis resistance. After tumor cells leave the primary tumors and invade into the blood circulation, they activate the platelets that encase them to protect them from the shear stress and immune attack. Platelets through the secretion of VEGF, PDGF, or TGF-β promote downregulation of NKG2D receptor and induce NK cells anergy. By arresting tumor cells at the vascular wall via P-selectin and its ligands, platelets facilitate extravasation and distant metastasis

Apoptosis is a programmed cell death (PCD) that occurs in response to irreparable DNA damage or induced by inflammatory cells. Anoikis is an apoptotic cell death triggered by the loss of ECM contact or inappropriate adhesion.^120^ Anoikis resistance is a crucial process that helps evade apoptosis of disseminating cells during metastasis and therefore promotes the development of aggressive tumors.^121^ Many studies have anticipated inhibitors of apoptosis (IAP) gene family as the most likely candidates conferring anoikis resistance in HNSCC.^122^ TIMP metallopeptidase inhibitor 1 (TIMP-1) exerts anti-apoptotic effects by activating FAK/PI3K and MAPK signaling pathways.^123^ However, recent studies have shown that TIMP-1 is downregulated in anoikis insensitive cells suggesting its role in regulating anoikis in HNSCC.^122^ Similarly, anoikis-resistant HNSCC JMAR cells grown under attached conditions show increased expression of paternally expressed gene 10 (PEG10 gene).^122^ This PEG10 protein has been shown to regulate apoptosis via interaction with E3 ligases, including seven in absentia homolog-1 and 2 (SIAH1 and 2) proteins.^122^ Further studies using these JMAR cells also showed increased expression of small proline-rich proteins (SPRK, SPRR 1A, SPRR1B, SPRR2A, and SPRR3) and S100 gene family calcium-binding secretory proteins (S100P, S100A7, and S100A9).^124^ Overexpression and activation of neurotrophic tyrosine kinase receptor-B (TrKB) was the first signaling pathway identified to facilitate anoikis resistance in HNSCC.^125^ Later, activation of integrins and their interactions with cytoplasmic kinases, small G-proteins, and scaffolding proteins mediated survival signaling pathways were found to be important for anoikis resistance.^126^ Tumor cells exhibiting decreased focal adhesion kinase (FAK) and increased p38 activation in the absence of adhesion undergo apoptosis.^127^ However, integrin-mediated activation of FAK/Src signaling has been shown to induce PI3K/AKT signaling and promote anoikis resistance in HNSCC.^126^ Additionally, hepatocyte growth factor (HGF) mediated activation of c-Met, PI3K/AKT/ERK, NF-κB signaling pathways have also been associated with anoikis resistance in HNSCC cells.^128,129^ It was recently shown that endothelins (secreted by endothelial cells) by promoting IL-6 and CCLX8 expression activates STAT3, PI3K/AKT/ERK pathways, and encourages anoikis resistance in HNSCC.^130^ Besides, secretion of VEGF by TME-associated endothelial cell-secreted factors also prevents anoikis via PI3K/AKT activation in HNSCC CSCs.^131^ In addition, EGF secretion by TAMs and endothelial cells via the ERK/c-Myc pathway induces angiopoietin-like 4 (ANGPTL4, secretory protein) expression which regulates NADPH oxidase 1 (Nox1) activation, regulates ROS levels and MMP expression. In the HNSCC cells, ANGPTL4 has been shown to activate the integrin β1 survival signaling pathway and protect the cells from anoikis.^132^ The formation of multicellular aggregates, also called emboli, is a crucial prerequisite to escape anoikis. Formation of a “platelet cloak” by tumor cell-induced platelet aggregation (TCIPA) shields the tumor cells from NK cell-mediated cytotoxicity and high shear forces in the bloodstream^133^ (Fig. 3). S100A7 or psoriasin is overexpressed in HNSCC, regulates the PKB/Akt signaling pathway, and induces anoikis resistance in HNSCC.^134^ Other studies reported the upregulation of S100P, KLK-related peptidase 6 (KLK6), and catenin alpha-like 1 (CTNNAL1) in the HNSCC anoikis-resistant cells compared to anoikis-sensitive cells, suggesting their role in the promotion of anoikis resistance.^122^ In addition to the secretory factors, many ECM protein fibronectin-mediated integrin αV-FAK activation has been shown to prevent p53-induced anoikis in HNSCC.^135^ Furthermore, another ECM protein collagen-I promotes tumor cell differentiation, invasion, migration and survival, and regulates anoikis resistance.^136,137^

### Role of HNSCC TME in angiogenesis

Tumor angiogenesis, the process of new blood vessel formation from pre-existing vessels, is critical for tumor growth and metastasis.^138^ Cytokines, chemokines, and growth factors, including VEGF, PDGF, and IL-8 by HNSCC cells and stromal cells, activate endothelial cells and induce angiogenesis.^131^ In particular, the interaction of endothelial and HNSCC cells triggers the NOTCH-1 signaling pathway and promotes capillary tubule formation. The endothelial cells secrete VEGF, which in an autocrine manner, activates VEGFR1 signaling and induces the expression of proangiogenic chemokines such as CXCL1, and CXCL8 thereby facilitating the proliferation of endothelial cells and angiogenesis.^139,140^ Under hypoxic conditions, TAMs by secreting TGF-β, VEGF, GM-CSF, TNF-α, IL-1, IL-6, and IL-8 stimulate angiogenesis. More specifically, IL-8, IL-6, and EGF induce phosphorylation of STAT3 and ERK in the endothelial cells resulting in their increased survival and proliferation to promote angiogenesis.^130^ CAF-secreted CXCL12 via CXCL12/CXCR4 signaling in endothelial cells controls angiogenesis by upregulating VEGF expression.^141^ TGF-β, which is present in the HNSCC TME and also secreted by Tregs, CAFs, TAMs, and MSDCs also increase angiogenesis.^142^ Similarly, lymphotoxin-α (LT-α), a member of the TNF superfamily, and an important pro-inflammatory cytokine secreted by B and T lymphocytes, increases angiogenesis by regulating CXCL2 expression. A recent study has shown that increased secretion of LT-α from HNSCC TILs induced angiogenesis via increasing endothelial PFKFB3 expression and enhanced glycolytic flux.^143^ Extracellular vesicles (EVs), secreted from many cell types, are known to mediate cell-to-cell communication by carrying signaling molecules, including VEGF, TGF-β, bFGF. Sato et al. showed that HNSCC cells secreted EVs carry ephrin type B receptor 2 (EPHB2), promoting angiogenesis by inducing the STAT3 signaling pathway in endothelial cells.^144^ Another study showed that tumor-derived exosomes (a form of EV) carry coagulation factor III, IGFBP-3, uPA, thrombospondin-1, endostatin, and uPA. The authors further demonstrated that through the uPA/uPAR system, exosomes promoted EC proliferation and migration and, thus, stimulate angiogenesis.^145^ Besides soluble factors, increased levels of nitric oxide (NO) have also shown to trigger neo-vascularization, thereby promoting tumor growth and metastasis.^146^

### Immune surveillance of HPV^+ve^ and HPV^−ve^ HNSCC tumors

Frequently formed premalignant lesions are eliminated by the immune system before the development of invasive tumors.^147^ However, tumor cells also acquire multiple mechanisms to evade the immune system. These include downregulation of major histocompatibility complex (MHC) class I or class II molecules, loss of immune costimulatory molecules and gain of co-inhibitory signals, defects in antigen-processing machinery (APM), as well as the presentation of tumor-associated antigens (TAA), and downregulation of TAA.^148^ In addition, overexpression of immunosuppressive molecules, including TGF-β, PGE2, IL-6, IL-10, adenosine, and/or the expression of Fas ligand (FasL), lead to the death of TILs^148^ and help cancer cell to escape immune recognition. Furthermore, the recruitment of immunosuppressive and tumor-promoting Tregs, MDSCs, TAMs cells, and dysfunction of antitumor immune cells produce tumor pervasive microenvironment and aid in developing clinically evident malignancies. In HNSCC, reduced expression and function of HLA- and APM and associated poor TA processing and presentation prevent NK activation and escape from T-cell-mediated lysis.^149^ Although the mechanism(s) of impaired APM expression is still unclear in HNSCC, EGFR-mediated HLA downregulation is essential for evading immune recognition.^150^ Also, overexpression of checkpoint inhibitors (ICIs) in the HNSCC TME like CTLA-4, PD-1, LAG3, TIM3, and killer immunoglobulin-like receptor (KIR) promote T-cell anergy or apoptosis and therefore fail to induce toxicity.^150^ Furthermore, downregulation of the classical costimulatory molecules CD28/B7 and other ligands CD137/CD137-L, OX40/OX40-L, and CD40/CD40-L observed in HNSCC is associated with poor patient outcome.^151^ High expression of TGF-β1 produced by HNSCC cells is known to downregulate CD16 and the NK cell receptor NKG2D associated with decreased biological functions of NK cells.^152,153^ Increased expression of FasL, TRAIL by HNSCC cells, or MAGE3/6^+^FasL^+^MHC class I^+^ exosomes derived from serum of HNSCC patients has been shown to induce T-cells apoptosis and suppress the immune response.^153,154^ High expression of the tryptophan-catabolizing enzyme indoleamine 2,3-dioxygenase (IDO) arrests the clonal expansion of T cells. Increased expression of IDO by the HNSCC cells, or by MDSCs and dendritic cells has been shown to reduce tryptophan levels needed for T-cell growth and the production of Granzyme B, thereby enhancing immune evasion.^155^ Moreover, increased expression of HLA-E by HNSCC cells that are known to inhibit NK and CD8^+^ T cells via NKG2A represents another mode for immunosuppression.^149^ Ectonucleotidases like CD39 and CD73 expressed by Tregs convert ATP into immunosuppressive adenosine. Increased expression of CD39, CD73, and adenosine in the HNSCC patients is thought to be associated with more potent Teff cell suppression.^153^ HNSCC tumors show an increased number of MDSCs,^156^ and by producing NO and ROS catalyze the nitration of the T-cell receptor, prevent TCR-HLA interaction and subsequent activation.^157^ HPV-associated tumors represent a significant proportion of HNSCCs with enhanced production of TGF-β, decreased expression of IFN-α, TLR9, low viremia, infection confined to keratinocytes with no cell lysis that prevents inflammation. Therefore, no inflammatory response is employed to escape immune surveillance against HPV.^153^ Although specific T cells against the oncogenic HPV E7 protein have been detected, these cells can not eliminate the tumors. Though the underlying reasons still need to be clarified, it has recently been shown that NLRX1-mediated degradation of proximal nucleic acid-sensing proteins such as the interferon gene stimulator (STING) complex promotes immune escape in HPV^+ve^ tumors.^158^

### Role of HNSCC TME in development of metastatic niche

The development of metastatic HNSCCs remains the primary cause of morbidity and mortality. The term metastatic niche (MN) refers to the well-suited microenvironmental conditions for tumor cells to survive and colonize in and disseminate to distant organs.^159^ The important components of the pre-metastatic niche include secreted factors from tumor cells (TDSFs), EVs, bone marrow-derived cells (BMDCs), suppressive immune cells, and host stromal cells.^159^ MN formation involves deposition and remodeling of ECM components, perivascular location and vascular remodeling, regulation of growth factors, cytokines, chemokines and MMPs expression, the establishment of inflammatory milieu and hypoxia, stemness, mesenchymal-to-epithelial transition (MET), and dormancy.^160^ While the formation of the primary tumor is dependent on progressive genetic changes in an inflammatory stroma, formation of niche either pre-metastatically or after the settlement of DTCs serves to re-establish the stromal environment required for metastatic tumor growth.^161^ Integrin-mediated interaction of DTCs with the modified niche ECM induces FAK signaling, promoting their proliferation and survival.^162^ Endothelial cells also play other roles in the development of perivascular microenvironment (ME) and MN. By secreting pro-inflammatory cytokines, S100A8 and S100A9, endothelial cells help in the recruitment of CTC and myeloid CD11b^+^ cells.^163^ Lysyl oxidase (LOX) and LOX-like proteins (LOXL) activity at these pre-metastatic sites help cross-link collagen IV and elastin that provide the substrate for CD11b^+^ cell attachment.^164^ The expression of S100A8 and S100A9 at the pre-metastatic niche (PMN) also promotes the secretion of serum amyloid A3 (SAA3) protein that helps to recruit myeloid cells,^160,163^ stimulate TNF-α expression and promote inflammatory milieu. Hypoxia is also a critical TME component promoting tumor cell dormancy and its dissemination. By inducing the expression of VEGF-A, placenta growth factor (PlGF), LOX, and stromal cell-derived factor (SDF1α), hypoxia helps in the recruitment of circular hematopoietic progenitor VEGFR1^+^ and CD11b^+^ BMDCs and remodeling of ECM components during the pre-metastatic niche formation. Along with CAF-secreted SDF1, VEGFR1^+^ BMDC recruit CXC4^+^ tumor cells to pre-metastatic niche. The CD11b^+^ cells by stimulating the versican (ECM proteoglycan) inhibit the TGF-β/Smad2 signaling pathway, promote MET, increase proliferation, and speed up metastasis.^165^ Both VEGFR1^+^ BMDC and CD11b^+^ secrete large amounts of MMP9, which in turn, participate in ECM and vascular remodeling, angiogenesis, and vasculogenesis.^160,166^ These VEGFR1^+^ BMDC cells directly recruit VEGFR2^+^ circulating endothelial progenitor cells and thereby help to vascularize the niche and promote tumor sprouting. HNSCC cell migration and metastasis are also promoted by the expression of macrophage inflammatory protein 3α (MIP-3α).^167^ At the metastatic niche, TAMs provide survival signals for VCAM-1-expressing tumor cells. The MCP-1 secreted by the tumor cells help recruit MDSC and NK to the MN. Also, tumor-secreted VEGFA stimulates TAMs to produce CXCL1 thereby recruiting MDSCs to PMN. After reaching the PMN, DTCs enter a dormant state to adopt the host microenvironment or proliferate to form multicellular micrometastases. This dormancy is reversed by the activation of MET, restore epithelial features, and help tumor relapse. The CD11b^+^ cells by stimulating versican (ECM proteoglycan) synthesis inhibit TGF-β/Smad2 signaling pathway, stimulate MET, increase proliferation, and promote metastasis.^165^

### TME promotes therapeutic resistance in HNSCC

Despite significant advances, resistance to therapy is still a challenge and the leading cause of poor prognosis of HNSCC patients. The mechanisms underlying resistance to CRT are very complicated and multifactorial. The genomic complexity, intratumoral genetic heterogeneity, and field cancerization result in the deregulated expression of drug target, drug transporters, pro-survival, and apoptotic pathways are considered important underlying reasons. More importantly, increased expression of transporters including ATP-binding cassette (ABC) family members, including P-glycoprotein (P-gp) drive expulsion of routinely used chemotherapeutic drugs (cisplatin, carboplatin, oxaliplatin, and 5FU) and is associated with multidrug resistance (MDR) in HNSCC. In addition to MDR, the deregulation of DNA repair signaling pathways, anti-apoptotic proteins (Bcl2, Bcl-xL, survivin, BMI), and tyrosine kinases (EGFR, cMET), also promote resistance to these DNA damaging agents.^168^ In addition to these acquired mechanisms, a growing body of evidence suggests that tumor-initiating cells (TICs) or CSCs are intrinsically resistant to standard CRT.^169,170^ All these mechanisms have been known for decades in HNSCC, but issues related to the resistance mechanism to IT have been gradually emphasized recently. These include lack of production, editing, and neo-antigen presentation, impaired immune infiltration, deregulation of immune checkpoints, T-cell exhaustion, and presence of suppressive immune cells (reviewed in ref. ^171^) The presence of Tregs and TAMs are significant contributors to the progression, therapeutic response, and resistance in many cancers, including HNSCC. While the recent preclinical studies showed increased Tregs population in immune ICIs and RT-treated recurrent tumors,^172^ the use of anti-CD25 antibody increased Teff/Treg ratio and improved ICI efficacy.^173^ Programmed death-ligand 1 (PD-L1), also known as cluster of differentiation 274 (CD274) or B7 homolog 1 (B7-H1) has recently been associated with resistance to immunotherapy and immune escape in multiple cancers, including HNSCC. Recent studies demonstrated increased expression of PD-L1 on CD68^+^ TAMs and tumor cells in the tonsillar crypts, the site of initial HPV infection. In addition, these studies also showed increased expression of PD-L1 inhibitor PD-1 on the CD8^+^ tumor-infiltrating lymphocytes (TILs).^174^ These studies suggest that tonsillar crypts are immune-privileged site with a downregulated effector function of virus-specific T cells and facilitating immune evasion of the HPV-infected cells. These findings are highly compatible with a model in which IFN-γ and potentially other cytokines associated with an immune response induce PD-L1 on tumor cells, which then downmodulates antitumor immunity to facilitate tumor survival. Thus, the PD-1: PD-L1 pathway plays a role in both persistence of HPV infection (through the expression of PD-L1) in the tonsillar crypt epithelium as well as resistance to immune elimination during malignant progression.^174^ In addition to inhibitory molecules, the overexpression of alternative co-inhibitory receptors like CTLA-4, TIM-3, OX40, lymphocyte-activation gene 3 (LAG3) is known to promote T-cell exhaustion and induces resistance to ICI.^175^ Besides, downregulation of human leukocyte antigen (HLA) class I molecules and loss of β2-microglobulin expression interfere with antigen presentation to cytotoxic T cells. Furthermore, loss of a classical tumor-suppressor gene PTEN induces expression of CCL2 and VEGF, thereby blocking T-cell infiltration and promoting resistance to the ICIs. Similarly, activation of β-catenin/WNT signaling decreases CCL4 production and inhibits the infiltration of DCs. It is essential to mention here that resistance to ICI is associated with an increased number of memory CD8 T cells (CCR7^−^CD45RA^−^), lower CD4/CD8 ratio, and increased expression of checkpoint inhibitor TIM-3 on CD4 as well as CD8 T cells.^176,177^

As noted earlier, hypoxia is a crucial component of HNSCC TME and plays a vital role in developing resistance to CRT and IT. Increased expression of hypoxia-inducible factors (HIF-1α and HIF-2α) has been shown to modulate both the innate and adaptive immune responses by affecting cytokine production from tumor and endothelial cells, thereby controlling the infiltration of T lymphocytes and macrophages into the tumors.^178^ Hypoxia induces endoplasmic reticulum (ER) stress response and promotes growth arrest and resistance (hypoxia-induced) to chemotherapy.

In addition to cytokines, chemokines, and growth factors, tumor and the stromal cells secrete many microRNAs (miRNAs) that control growth, proliferation, EMT, angiogenesis, immune surveillance, and impart CRT resistance in HNSCC. While many miRNAs are differentially expressed in HNSCC compared to normal tissues,^179^ many recent studies have shown the differential miRNA expression profile among HPV^+ve^ and HPV^−ve^ HNSCC.^180,181^ These studies have shown the association of core miRNAs, including miR-15a/miR-16/miR-195/miR-497 family, miR-143/miR-145, and the miR-106–363 with HPV infection in HNSCC.^180^ While the expression of miR-195, miR-497, miR-143, miR-145, miR-199a-3p, miR-199b-3p, miR-199b-5p, and miR-126 was significantly downregulated, miR-15a, miR-16, miR-363, miR-363, miR-20b, and miR106b~25 were found to be upregulated in HPV^+ve^ HNSCC patients.^180,182^ Using the microarray analysis, miRNA-363 and miRNA-33 were found upregulated, whereas miRNA-155, miRNA-181a, miRNA-181b, miRNA-29a, miRNA-218, miRNA-222, miRNA-221, miRNA-42-5p, and miRNA-1323 were downregulated in HPV^+ve^ SCC2 and SCC90 cells compared to HPV^−ve^ PCI13 and PCI30 cells.^183^

Interestingly, significant upregulation of miR-101, miR-181d, miR-181b, and miR-195 and downregulation of miR-100, miR-130a, and miR-197 has been reported in drug-resistant HNSCC cells compared to their parent cells.^184^ Similar overexpression of miRNA-23a, miRNA-214, miRNA-518c, miRNA-608, let-7 family of miRNAs, and downregulation of miRNA-21 and miRNA-342 were observed in cisplatin-resistant TSCC compared to sensitive cells.^185^ Though the underlying reasons for imparting cisplatin resistance are entirely unknown, miRNA-214 mediated PTEN decay, thereby PI3K/Akt signaling pathway activation, is considered one of the mechanisms.^185^ Overexpression of miR-125a promotes cisplatin sensitivity in laryngeal CSCs by targeting hematopoietic cell-specific protein 1-associated protein X-1 (HAX-1).^186^ Furthermore, miR-125a-mediated HAX-1 downregulation also promoted vincristine, etoposide, and doxorubicin sensitivity in laryngeal CSCs.^186^ Importantly, overexpression of miR-222^187^ and miR-24-3p^188^ promoted resistance to chemotherapeutic drugs via targeting ABCG2 and CHD5, respectively.

Compared to miRNAs involved in CT resistance, many miRNAs that regulate radio-resistance (RR) have been found. For example, miR-296-5p is downregulated in LSCC and its upregulation in enhanced radiosensitivity (RS) by targeting MDR1 gene.^189^ Similarly, miR-125b is downregulated in OSCC, but its overexpression enhanced RS via ICAM2 downregulation and PI3K/Akt signaling pathway activation.^190^ Both the miR-324-3p^191^ and miR-185-3p^192^ are downregulated in the RR NPC cells, and their upregulation promoted radiosensitivity via targeting WNT2B gene. Similarly, miR-196a^193^ and miR-451 determine RR via targeting annexin-1 (ANXA1) and RAS related protein 14 (RAB14), respectively, in HNSCC.^194^ Similar to miRNA-214, miR-205^195^ and miR-296-5p^196^ via targeting PTEN promote RR in NPC. Furthermore, miR-23a^197^ and miR-203^198^ play an important role in radiosensitivity by targeting IL-8 in NPC. While the pathobiological importance of many of these miRNAs is known in HNSCC, limited studies are available showing their role in imparting CRT resistance specific to HPV^+ve^ HNSCC.

## Targeting HNSCC TME: differential response among HPV^+ve^ and HPV^−ve^ tumors

**Fig. 4:**
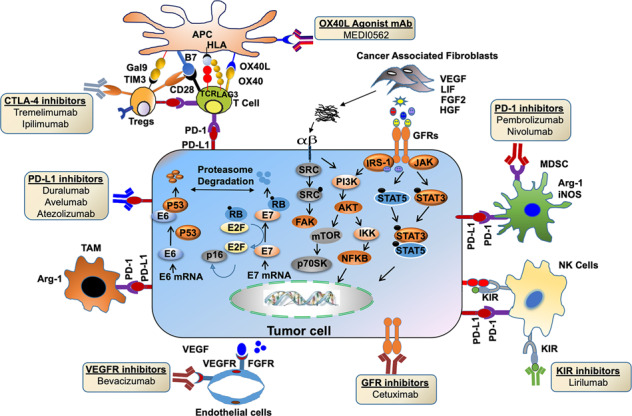
Schematic showing the immunotherapeutic approaches targeting the HNSCC TME. Inactivation of CTLs by secretory immunomodulators or immune checkpoints promotes immune escape of cancer cells. While B7 ligands on APCs interact with CD28 on CTL and provide a secondary signal for their activation and immune response, CTLA-4 on Tregs interfere in this interaction, suppresses CTLs activity and enhances Treg activity. In addition, increased PD-L1 on cancer cells or immune cells like NK, MDSCs, M2 macrophages, and Tregs bind to its receptor PD-1 on activated T cells to promote the state of anergy in CTLs. The OX40 ligand on the APC cells interacts with the OX40 receptor on T cells and increase CTL activation. Cancer cells secrete VEGF, which, upon binding with its receptor VEGFR on endothelial cells, promotes angiogenesis. Many pharmacological inhibitors, including monoclonal antibodies/agonists of immune checkpoints, are being tested in HNSCC and shown to restore CTL antitumor activity and relieve immunosuppression

Tumor progression, metastasis, and response to therapy are markedly affected by the complex heterotypic multicellular interactions among tumor and other cellular and noncellular components of TME. The presence of dense stroma also limits drug availability and compromises therapeutic efficacy. HPV^+ve^ tumors are immune-rich and more responsive to CRT with significant locoregional control^199,200^ than HPV^−ve^ tumors.^201^ However, recent studies have shown that increased expression of PD-L1 and PD-L2 on fibroblasts and tumor cells make these tumors immunosuppressive.^202^ Many immunotherapies that target immune checkpoints like PD-1/PD-L1 and CTLA-4 are currently being evaluated. Several mAbs targeting these ICIs are currently being investigated to increase cancer-specific immunity in HNSCC (Table 2 and Fig. 4). However, differential mutational burden and immunologic landscape among HPV^+ve^ and HPV^−ve^ HNSCCs result in disparate response to therapy and prognosis. PD-L1 is a ligand for PD-1 and PD-2 receptors that limit T-cell activation during inflammation and restrict autoimmunity. PD-L1 is overexpressed in 66% of HNSCC patients and more often in HPV^+ve^ than HPV^−ve^ patients. Recently, fully humanized anti-PD-L1 mAb (durvalumab) was evaluated in the HAWK study on platinum-refractory (PR) R/M HNSCC patients.^203^ The interim results from this study showed a higher overall response rate (ORR) in HPV^+ve^ patients than HPV^−ve^ patients (30% *vs* 10%).^203^ Similarly, the use of FDA approved pembrolizumab (anti-PD-1)^204^ and nivolumab (anti-PD-1)^205^ mAbs in PR R/M HNSCC patients showed improved OS in HPV^+ve^ HNSCC patients compared to HPV^−ve^ patients. Combination therapies targeting both the inhibitory and costimulatory receptors have synergistically enhanced the immunological antitumor effects. The dual-blockade therapy combining durvalumab with tremelimumab (anti-CTLA-4 mAb) is currently being investigated for R/M HNSCC (NCT02551159, NCT02369874, and NCT02319044). Similarly, nivolumab in combination with ipilimumab (an IgG1 mAb against CTLA-4) and standard therapy cetuximab/platinum/5FU (NCT02741570) or with ulocuplumab (a fully human anti-CXCR4, NCT02472977) is also being investigated in LA/M HNSCC tumors. Cetuximab, is a chimeric murine/human IgG1 monoclonal antibody (mAb) against EGFR, and is the Food and Drug Administration (FDA) approved targeted agent for the treatment of HNSCC. Triple combination therapy involving nivolumab, ipilimumab, and anti-LAG3 mAb (BMS-986016) is also being investigated in R/M HNSCC patients (NCT02488759). Agonistic anti-OX40 and anti-CD27 agents activate OX40 and CD27, respectively, and both promote T-cell proliferation. In this direction, clinical trials to assess the safety and tolerability of durvalumab along with agonistic anti-OX40 Ab (NCT02221960), and varlilumab with agonistic anti-CD27 mAb (NCT02335918) are currently ongoing in HNSCC patients.

Besides inducing tumor cell death, the standard-of-care CRT promote antitumoral immune responses by depleting and exhaustion of CTLs, development of CRT-resistant Tregs, increasing MDSCs and enhancing tumor cell MHC I expression.^206^ However, recent studies have shown that lower doses of CRT also trigger adaptive and innate immune response^207^ and improve immune recognition of dying cancer cells by mobilizing bone marrow hematopoietic and myeloid cells, increasing infiltration of DCs, CD4^+^, and CD8^+^ T cells^208^ associated with IFN-γ and IL-2 secretion,^209^ and elimination of MDSCs.^210^ These studies suggest that the combination of lower CT or RT doses and ICI will improve response in patients with poor immune-infiltrated tumors. Cetuximab administration is associated with decreased tumor cell proliferation, inhibition of angiogenesis, and reduced metastasis. It also binds to the CD16 receptor on NK cells and DCs and improves immune response by promoting T-cell priming, ADCC, and NK cell activation associated with increased HNSCC patient survival.^211^ However, the limited survival benefits of cetuximab are linked to increased expression of CTLA-4 by Tregs associated with poor prognosis in HNSCC patients.^212^ In this context, the use of ipilimumab was shown to reverse cetuximab resistance in HNSCC by activating NK cells thereby eliminating Tregs.^212^ A combination of pembrolizumab with platinum-based CT and 5FU with or without cetuximab is currently being investigated in HNSCCs (NCT02358031). RT is known to increase PD-L1 expression rendering HNSCC TME immunosuppressive.^213^ Based on the diverse immunomodulatory effects of RT, the combination of ICI with RT is under intense investigation. Many clinical trials combining ICI including nivolumab with stereotactic body radiotherapy (STBR) (NCT02684253) or intensity-modulated radiation therapy (IMRT) along with chemotherapeutic agents like cisplatin (NCT02764593) have recently been started. A combination of ipilimumab with IMRT or with nivolumab or cetuximab is currently being established in HNSCC (NCT01860430, NCT02741570, and NCT01935921). In addition, efficacy of concurrent vs sequential pembrolizumab, cisplatin, and IMRT is being evaluated in HNSCC (NCT02777385). Besides targeting immune cells, clinical trial for use of humanized IgG1 anti-PD-L1 mAb atezolizumab along with bevacizumab (mAb against VEGF) is currently enrolling HPV or EBV-associated HNSCC patients (NCT03074513). Similarly, use of FAP (CAF-specific protein) through PT100 combined with oxaliplatin improved CT, reduced recruitment of MDSCs, Tregs, and inhibited angiogenesis.^214^ Besides, inhibition of CAF promoted CXCL12/CXCR4 signaling using CXCR4 antagonist (TN14003), or with the anti-CXCR4 antibody AMD3100, or siRNAs inhibited growth and invasion in vitro.^111,215–217^

Furthermore, combining ulocuplumab (a fully human anti-CXCR4) with nivolumab is currently being investigated for LA/M solid tumors (NCT02472977). Similarly, a combination of cell-cycle regulator CDK4/6 inhibitors (abemaciclib and palbociclib) and PD-L1 inhibitor avelumab has been started in HNSCC (NCT03498378). Keeping in view the enhanced efficacy of CDK4/6 inhibitors in RB-positive tumors, HPV^+ve^ HNSCCs may respond more effectively to this combination owing to their high RB and PD-L1 expression. As mentioned previously, HPV^+ve^ tumors exhibit increased glycolysis and OXPHOS.^55^ Use of mTOR (regulator of OXPHOS) inhibitor rapamycin along with anti-PD-L1 mAb showed improved survival by increasing IFN-γ production in tumor-infiltrating CD8^+^ T cells.^218^ In addition to these studies, several other clinical trials using combinations of ICI and CRT are underway and included in Table 2.

Epidermal growth factor receptor (EGFR), a transmembrane tyrosine kinase receptor (TKR) of the ErbB family, is overexpressed or amplified in ~90% of HNSCC patients^219,220^ and is associated with aggressive tumor behavior,^221^ resistance to radiation,^222^ and poor prognosis.^223,224^ The constitutive activation of EGFR result in the activation of several downstream signaling pathways, including Ras/Raf/MAPK/ERK, PI3K/Akt, STAT, and the PLC-γ signaling pathways,^225,226^ those are involved in proliferation, survival, CSCs maintenance^227^ etc. While the relationship between HPV status, EGFR expression and patient outcome are still ambiguous in HNSCC, many studies have reported increased EGFR amplification in p16^−ve^ (a surrogate biomarker for HPV infection) oropharyngeal cancers.^228,229^ In this direction, several studies have conclusively established better clinical outcomes in HPV^+ve^ HNSCC patients with low EGFR expression.^229–232^ Interestingly, the use of cetuximab against EGFR is the first FDA approved targeted therapeutics in HNSCC. However, constitutively active EGFR variant (EGFRvIII) promotes resistance against cetuximab in HNSCC.^233^ While the therapeutic efficacy of EGFR inhibition remains unclear and controversial in HPV^+ve^ HNSCC,^234^ many other EGFR inhibitors including mabs (panitumumab or nimotuzumab) and small-molecule inhibitors (lapatinib, erlotinib, gefitinib, and afatinib), either alone or along with CRT are currently being investigated in many clinical trials (reviewed in ref. ^235^).

## Conclusion and future perspective

The primary goal of HNSCC treatment is to inhibit growth and prevent metastasis. However, most of the previous preclinical studies based more on aberrant genetic and epigenetic mutations, altered gene expression in tumor cells have failed in clinical trials. The underlying reasons being the limited understanding of the (1) HNSCC tumor biology and (2) the importance of TME in the progression and development of therapeutic resistance. Recent clinical, genomic, and cellular studies have demonstrated HNSCC TME as highly heterogeneous and immunosuppressive. Although the current clinical trials involving the combination of CT or RT with immune checkpoint blockers are underway, low ORR warrants a better understanding of the role of the immune system in HNSCC. Many recent studies have shown the dynamic nature of the TME during tumor progression or upon the administration of therapeutic interventions. High-throughput analysis should be utilized to comprehensively investigate the spatiotemporal, in-depth characterization of phenotypic, functional features of diverse cell types, and their dynamic cross talk in HNSCC TME during tumor progression and metastasis.

The emerging knowledge of the differential molecular and immune landscape between HPV^+ve^ and HPV^−ve^ HNSCC tumors has created newer opportunities to develop a personalized, targeted CIT approach. Therefore, designing of CIT trials based on genetic, molecular, and immunological landscape and topography of immune cell distribution in the TME will help develop new strategies for effective antitumor immunity and improved clinical outcomes. Future rational combination studies should consider the impact of biological and mechanistic cross talk between cancer cells—-TME—-and therefore include coculture, organoids/tumoroids, and humanized patient-derived xenograft (PDX) models in preclinical studies. Besides, the use of immunocompetent animal models ((Kras^G12D^;Trp53^R172H/+^;K14-CreER^tam^) and E6/E7;Kras^G12D^;K14-CreER^tam^)) that histopathologically recapitulate the tobacco/alcohol^236^ and HPV-mediated^237^ human HNSCC pathogenesis, respectively, should be considered for developing CITs. Considering the importance of HPV infection on immune HNSCC milieu and, therefore CIT response, future clinical trials should take into account HPV status and immune phenotype to escalate a precision regimen to clinics and ensure better therapeutic outcomes in HNSCC patients. While the development of therapeutic resistance is unavoidable, identification of reliable response markers and understanding resistance is essential.
