# Correction: Tumor microenvironment: an evil nexus promoting aggressive head and neck squamous cell carcinoma and avenue for targeted therapy

**DOI:** 10.1038/s41392-021-00503-9

**Published:** 2021-02-26

**Authors:** Ajaz A. Bhat, Parvaiz Yousuf, Nissar A. Wani, Arshi Rizwan, Shyam S. Chauhan, Mushtaq A. Siddiqi, Davide Bedognetti, Wael El-Rifai, Michael P. Frenneaux, Surinder K. Batra, Mohammad Haris, Muzafar A. Macha

**Affiliations:** 1 Functional and Molecular Imaging Laboratory, Cancer Research Department, Sidra Medicine, Doha, Qatar; 2 Department of Zoology, School of Life Sciences, Central University of Kashmir, Ganderbal, Jammu & Kashmir India; 3 Department of Biotechnology, School of Life Sciences, Central University of Kashmir, Ganderbal, Jammu & Kashmir India; 4 Department of Nephrology, All India Institute of Medical Sciences, New Delhi, India; 5 Department of Biochemistry, All India Institute of Medical Sciences, New Delhi, India; 6 Watson-Crick Centre for Molecular Medicine, Islamic University of Science and Technology, Awantipora, Jammu & Kashmir India; 7 Laboratory of Cancer Immunogenomics, Cancer Research Department, Sidra Medicine, Doha, Qatar; 8 Department of Surgery, University of Miami, Miami, FL USA; 9 Academic Health System, Hamad Medical Corporation, Doha, Qatar; 10 Department of Biochemistry and Molecular Biology, University of Nebraska Medical Center, Omaha, NE USA; 11 Eppley Institute for Research in Cancer and Allied Diseases, University of Nebraska Medical Center, Omaha, NE USA; 12 Buffett Cancer Center, University of Nebraska Medical Center, Omaha, NE USA; 13 Laboratory Animal Research Center, Qatar University, Doha, Qatar

**Keywords:** Head and neck cancer, Cancer microenvironment

Correction to: *Signal Transduction and Targeted Therapy* 10.1038/s41392-020-00419-w, published online 12 January 2021

After online publication of the article^1^, the authors noticed an error in affiliation of co-authors Dr. Nissar A. Wani. Dr. Wani is erroneously linked to Sidra Medicine, Doha, Qatar who is actually working at the Department of Biotechnology, School of Life Sciences, Central University of Kashmir, Ganderbal, Jammu & Kashmir, India. Also, the department name “Cancer Research Department” for the first author Ajaz A. Bhat is missing.
